# Enhanced biological activity of liposomal methylated resveratrol analog 3′-hydroxy-3,4,5,4′-tetramethoxystilbene (DMU-214) in 3D patient-derived ovarian cancer model

**DOI:** 10.1080/10717544.2022.2103210

**Published:** 2022-07-27

**Authors:** Andrzej Nowicki, Dariusz Wawrzyniak, Mikołaj Czajkowski, Małgorzata Józkowiak, Michał Pawlak, Marcin Wierzchowski, Katarzyna Rolle, Paulina Skupin-Mrugalska, Hanna Piotrowska-Kempisty

**Affiliations:** a Department of Toxicology, Poznan University of Medical Sciences, Poznan, Poland; b Department of Molecular Neurooncology, Institute of Bioorganic Chemistry, Polish Academy of Sciences, Poznan, Poland; c Department of Inorganic & Analytical Chemistry, Collegium Pharmaceuticum, Poznan University of Medical Sciences, Poznan, Poland; d Greater Poland Cancer Centre, Poznan, Poland; e Department of Chemical Technology of Drugs, Poznan University of Medical Sciences, Poznan, PL, Poland; f Department of Basic and Preclinical Sciences, Institute of Veterinary Medicine, Nicolaus Copernicus University in Torun, Torun, Poland

**Keywords:** Resveratrol, methylated resveratrol analogs, DMU-214, liposomes, ovarian cancer, tumor spheroids

## Abstract

3′-hydroxy-3,4,5,4′-tetramethoxystilbene (DMU-214) belongs to methoxystilbenes family and is an active metabolite of 3,4,5,4′-tetramethoxystilbene (DMU-212). In several of our previous studies, the anti-apoptotic activity of DMU-214 was significantly higher than that of the parent compound, especially in ovarian cancer cells. Due to increased lipophilicity and limited solubility, methoxystilbenes require a solubilization strategy enabling DMU-214 administration to the aqueous environment. In this study, DMU-214-loaded liposomes were developed for the first time, and its antitumor activity was tested in the ovarian cancer model.

First, several liposomal formulations of DMU-214 were obtained by the thin lipid film hydration method followed by extrusion and then characterized. The diameter of the resulting vesicles was in the range of 118.0-155.5 nm, and samples presented monodisperse size distribution. The release of DMU-214 from the studied liposomes was governed by the contribution of two mechanisms, Fickian diffusion and liposome relaxation.

Subsequently, *in vitro* activity of DMU-214 in the form of a free compound or liposome-bound was studied, including commercial cell line SK-OV-3 and patient-derived ovarian cancer cells in monolayer and spheroid cell culture models. DMU-214 liposomal formulations were found to be more potent (had lower IC_50_ values) than the free DMU-214 both in the monolayer and, more significantly, in both examined spheroid models. The above results, with particular emphasis on the patient-derived ovarian cancer model, indicate the importance of further development of liposomal DMU-214 as a potential anticancer formulation for ovarian cancer treatment.

## Graphical Abstract

**Figure:**
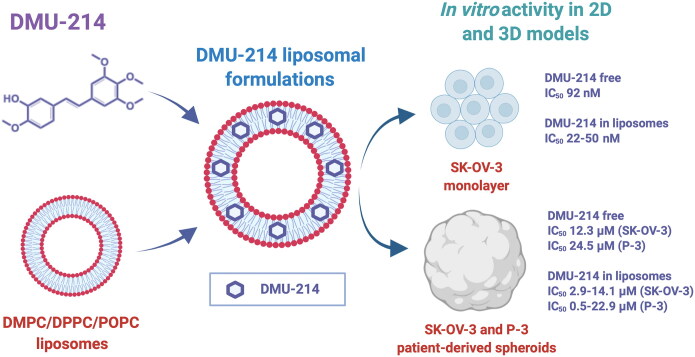

## 1. Introduction

Ovarian cancer represents the eighth most frequently diagnosed tumor and the seventh most lethal cancer in women worldwide (Bray et al., 2018; Falzone et al., 2021). Despite improved screening and the advancement of anticancer surgical and pharmacological treatments, ovarian cancer remains one of the most commonly diagnosed and aggressive female tumors of the genitourinary system (Falzone et al., 2018; Torre et al., 2018; Falzone et al., 2021). Several chemotherapeutic agents have been used to treat ovarian cancer. Recent progress in molecular therapies improved therapeutic outcomes in different histological and molecular subtypes of ovarian cancer and, consequently, patients’ quality of life and life expectancy (Shimada et al., 2017; Falzone et al., 2018; 2021). Still, the individual response to standard chemotherapy differs among patients and the prognosis of patients with ovarian cancer is often poor. Also, the treatment of advanced or metastatic ovarian cancer is challenging. Therefore, novel, effective personalized therapies based on patients’ molecular and genetic profiling are needed (Falzone et al., 2021). Currently, several approaches are being developed, including personalized therapy, therapeutic vaccines, adoptive cellular therapy, and T-cell therapy (Falzone et al., 2021). To achieve effective cancer therapies, more realistic models based on the biological characteristics of the individual patient are essential for predicting the response to therapy (Falzone et al., 2018).

Resveratrol (3,5,4′-trihydroxystilbene, RSV) was first isolated from the roots of white hellebore (*Veratrum grandiflorum* Loes fil.) in 1940 (Takaoka, 1939). RSV attracted little interest until 1992, when it was postulated to explain some of the cardioprotective effects of red wine (Siemann & Creasy, 1992). Since then, dozens of reports have shown that RSV can prevent or slow the progression of a wide variety of illnesses, including cancer (Jang et al., 1997), cardiovascular disease (Cho et al., 2017) and ischemic injuries (Sinha et al., 2002; Wang et al., 2002). In particular, over the past decades, RSV has been used as a dietary supplement possessing a broad spectrum of pharmacological properties (Zhang et al., 2021). However, Walle et al. (Walle et al., 2004) reported high absorption but poor systemic bioavailability resulting from the rapid metabolism of RSV when administered orally to humans. Due to the limited solubility of RSV in aqueous media (<0.001 mol/l) and, consequently, in biological fluids, high doses of RSV are required to reach beneficial effects (Bonechi et al., 2012). Several reports have demonstrated that the methylation of RSV enhances its bioavailability and bioactivity (Tolomeo et al., 2005; Mikstacka et al., 2007; Kang et al., 2014). Among several methoxystilbenes studied, 3,4,5,4′-tetramethoxystilbene (DMU-212) showed the most potent cytotoxicity, which exerted pro-apoptotic activity in several cancer cell lines, including transformed fibroblasts, liver, colon, hypopharynx, breast, prostate, and ovarian ones (Gosslau et al., 2005; Ma et al., 2008; Piotrowska et al., 2012; 2013). Furthermore, the cytotoxicity of one of the DMU-212 metabolites, 3′-hydroxy-3,4,5,4′-tetramethoxystilbene (DMU-214), was found to be significantly higher than that of the parent compound in ovarian cancer cells. Furthermore, DMU-214 was shown to trigger G_2_/M cell cycle arrest and receptor-mediated apoptosis in the SK-OV-3 ovarian cancer cell line lacking p53 (Piotrowska-Kempisty et al., 2016). Additionally, our latest study demonstrated for the first time that DMU-214 displays anti-migratory and antiproliferative activity in SK-OV-3 ovarian cancer cells (Nowicki et al., 2020).

Noteworthy, the substitution of the hydroxyl groups with the methoxy groups increases the lipophilicity of methoxystilbens over the RSV, which results in higher bioavailability, but simultaneously affects the solubility of methoxy derivatives (Kang et al., 2014). There have been attempts to overcome these limitations (e.g. aqueous solubility), particularly by using nano-based drug delivery systems (Sindhu et al., 2021). Nanoencapsulation has been proved effective in increasing the aqueous solubility, chemical stability, and bioavailability of many bioactive compounds (Coimbra et al., 2011), including RSV (Bonechi et al., 2012; Isailović et al., 2013; Zu et al., 2018). Among many drug delivery systems, liposomes are widely accepted biocompatible carriers that can be loaded with compounds of different lipophilic-hydrophilic nature and whose properties can be easily designed by the careful selection of lipid components (Allen & Cullis, 2013; Crommelin et al., 2020).

The main goal of the presented study was to design and characterize liposomal carriers for DMU-214. Further, we aimed to evaluate the biological activity of liposome-loaded DMU-214 in commercial and patient-derived ovarian cancer cell lines, involving 2- and −3 dimensional models.

## 2. Materials and methods

### 2.1. Materials

DMU-214 was synthesized as described elsewhere (Androutsopoulos et al., 2011). The identity and purity of the compound were confirmed using NMR and LC–MS. 1,2-dimyristoyl-*sn*-glycero-3-phosphocholine (DMPC, 14:0 PC), 1,2-dipalmitoyl-*sn*-glycero-3-phosphocholine (DPPC, 16:0 PC) were kindly provided by Lipoid GmbH Ludwigshafen am Rhein, Germany. 1-palmitoyl-2-oleoyl-*sn*-glycero-3-phosphocholine (POPC, 16:0-18:1 PC), 1-palmitoyl-2-oleoyl-*sn*-glycero-3-phospho-(1′-rac-glycerol) sodium salt (POPG) were purchased from Avanti Polar Lipids Inc. (Alabaster, AL, USA).

### 2.2. Liposome preparation and characteristics

DMU-214-loaded liposomes were prepared by a thin lipid film hydration method followed by extrusion as described here (Skupin-Mrugalska et al., 2021) by using chloroform solutions of DMPC, DPPC, POPC (50 mg/ml), POPG (50 mg/ml), DMU-214 (10 mg/ml). DMU-214 was loaded passively into vesicles during hydration. Appropriate volumes of stock solutions were mixed, and then chloroform was evaporated under gradually reduced pressure at 40 °C in a round-bottomed flask. The resulting lipid film was then hydrated in PBS buffer pH 7.4. The resulting liposome suspension was passed 21 times through the polycarbonate membrane (Whatman, Kent, UK) with pore diameters of 100 nm using a syringe extruder (Avanti Polar Lipids Inc., Alabaster, AL, USA). Unbound material was separated from liposomes by fast ultrafiltration using Amicon Ultra 2 ml centrifugal filters with molecular weight cutoff (MWCO) 50 kDa (Merck KGa, Darmstadt, Germany). The amount of DMU-214 incorporated into liposomes was determined by the chromatographic method (paragraph 2.5). Encapsulation efficiency EE (%) was calculated according to Eq. (1): EE (%) = (C_m_/C_i_) x 100 (1), where C_m_ is the concentration of DMU-214 loaded into liposomes, determined by HPLC, C_i_ is the initial (maximum) concentration of DMU-214 added in the liposomal formulation. The experiment was performed in triplicates.

### 2.3. Dynamic light scattering

The mean size and zeta potential of liposomes were determined by DLS using Malvern Zetasizer Nano ZS (Malvern Instruments Ltd., Malvern, UK). Measurements were carried out at 37 °C in disposable folded capillary cells. Per sample, ten measurements were done with a data acquisition time of 10 s each. Measurements were repeated three times. Before the measurements, liposome samples were diluted ten times in PBS.

### 2.4. In vitro release of DMU-214 from liposomes

*In vitro* release of DMU-214 from liposomes was performed by the dialysis method in 10 mM PBS (pH 7.4). 1% SDS (m/v) was added to provide sink conditions. 1 mL of the liposomal formulation was added to a dialysis bag (10 kDa MWCO, Spectra/Por, Spectrum Laboratories, Inc., Piscataway, NJ, USA) and immersed into 45 mL of release medium (*n* = 3) in 50 mL tubes. The tubes were protected from light and shaken at 150 rpm and 37 °C using an orbital incubation shaker IST3075 703 JEIO TECH (Lab Companion, Daejeon, Republic of Korea). 0.5 mL samples were withdrawn from the release medium and diluted twice in methanol at predetermined time points. The experiment was performed in triplicates. The release kinetics profiles were presented as a ratio of drug released/drug added to dialysis bags against time. The kinetics of DMU-214 release from liposomes were determined using DDSolver software by fitting obtained results to different kinetic models: Higuchi, Krosmeyer-Peppas, Peppas-Sahlin (Zhang et al., 2010).

### 2.5. HPLC/DAD analysis

To estimate the encapsulation efficiency of DMU-214 and release kinetics from liposomes, collected samples were analyzed by high-performance liquid chromatography (HPLC) equipped with a diode array detector (DAD) using Agilent 1200 system (Agilent Technologies Inc., Santa Clara, CA, USA). Separation was performed in Luna NH_2_ column – C18, 5 μm particle size, 100 Å, 4.6 × 250 mm (Phenomenex. Torrance, CA, USA). Isocratic HPLC measurements were performed at room temperature and a detection wavelength of 273 nm. The mobile phase consisted of 0.5% formic acid and methanol 45:55 (*v/v*) at the flow rate of 1 ml/min. To estimate the concentration of DMU-214 in analyzed samples, an appropriate calibration curve was established (R ≥ 0.999).

### 2.6. Monolayer (2D) cell culture and cell viability assays

SK-OV-3 ovarian cancer cell line was purchased from the European Type Culture Collection (Sigma-Aldrich Co., St. Louis, MO). The cells were maintained in phenol red-free DMEM medium Sigma-Aldrich Co. (St. Louis, MO) supplemented with 10% fetal bovine serum (FBS), 2 mM glutamine, penicillin (100 U/ml), and streptomycin (0.1 mg/ml) (Sigma-Aldrich Co., St. Louis, MO). SKOV-3 cells were maintained under the standard conditions at 37 °C in a humidified atmosphere containing 5% CO_2_. To determine the effects of DMU-214 (free compound and liposome-loaded) on cell viability, confluent stock cultures were harvested using trypsin-EDTA solution (Sigma-Aldrich Co., St. Louis, MO) seeded in 96-well plates at a density of 2 × 10^4^ cells/well, which was determined by Bürker chamber (Gunetti et al., 2012; Piotrowska et al., 2012). They were allowed to attach overnight. Tested compounds were added in the concentration range of 0-10 µM. DMU-214 was added from the stock solution prepared in DMSO. The final concentration of DMSO in cell treatment solutions was less than 0.1%. Control cells were cultured under the same conditions with 0.1% DMSO. After 72 h, the cell viability was determined spectrophotometrically using MTT (Sigma-Aldrich Co., St. Louis, MO) as a substrate, as described elsewhere (Fresta et al., 2020; Skupin-Mrugalska et al., 2021; Zhou et al., 2022). Briefly, the cells were incubated for 4 h at 37 °C with the mixture of DMEM and MTT (5 mg/mL), and the formed formazan crystals were dissolved by adding 200 µL of DMSO. Optical density was measured at the wavelength of 570 nm using an Elx-800 plate spectrophotometer (BioTek, Winooski, VT, USA).

### 2.7. Scaffold-free 3D spheroid cultures

The SK-OV-3 cell line and a patient-derived (P-3) cell line were used in spheroid culture studies.

#### 2.7.1. Sample collection and tissue processing for tumor spheroids preparation

The collection of ovarian cancer samples was performed at the Greater Poland Cancer Center (GPCC) with the approval of the institutional ethics committee (Approval No. 289/21). This study was conducted following all relevant guidelines and regulations. All patients participating in this study signed informed consent forms. Ovarian cancer tissue was collected during surgery, and samples were stored in a MACS® Tissue Storage Solution (Miltenyi Biotec) at 4 °C during transportation to the laboratory. The sample delivery time was 45–60 min. The tissue samples were set on a sterile petri dish on crushed ice on arrival in the laboratory. Subsequently, the cancer tissue was dissected to a 5 mm square under sterile conditions. The single-cell suspension from tumors was prepared using Human Tumor Dissociation kit (Miltenyi Biotec) and gentleMACS Dissociator, according to the manufacturer’s instructions. Briefly, cut tumors were put into C-tube (Miltenyi Biotec) with 5 ml/tumor of digestion buffer from the Human Tumor Dissociation kit. C- tubes were placed on the gentleMACS Dissociator, and a digesting program at 37° C was used. Post-digestion, cell suspensions were filtered with a 40 µm cell strainer. In case of a visible red pellet, erythrocytes were lysed in Red Blood Cell Lysis Solution (Miltenyi Biotec) for 2 min at room temperature, followed by two wash steps with 45 ml of PBS and centrifugation at 300 × g for 5 min. After centrifugation, cells were resuspended in DMEM/F12 (ThermoFischer Scientific) medium supplemented with 1 × GlutaMAX-I (ThermoFischer Scientific), 1 × B27 supplement (ThermoFischer Scientific), 1 × N-2 supplement (ThermoFischer Scientific), 50 ng/ml recombinant human FGF-2 (ThermoFischer Scientific), 50 ng/ml recombinant human EGF (ThermoFischer Scientific), 2.5 µg/ml insulin (Sigma-Aldrich), 100 U/ml penicillin (Sigma-Aldrich), 0.1 mg/ml streptomycin (Sigma-Aldrich).

#### 2.7.2. Spheroid preparation

The spheroid culture was provided using an ultra-low attachment plate (ULA). For cytotoxicity studies of DMU-214 in liposomal carriers, we used commercially available 96-well Nunclon Sphera-Treated U-shaped ULA culture dishes (ThermoFischer Scientific). Tumor spheroids from SK-OV-3 and patient-derived (P-3) cell lines were generated by seeding 5000 cells/well with a volume of 100 µl. Then plates were centrifuged (2 min, 800 rpm) and cultured for 7 days at standard cell culture conditions.

#### 2.7.3. Cytotoxicity in 3D cell cultures

The viability of treated tumor cells in the spheroid model was measured using the CellTiter-Glo 3 D cell viability assay (Promega, Madison, WI, USA). Briefly, into treated (72 h) and untreated tumor cells, an equal volume of reagent was added and incubated for 30 min. Then, the lysates were transferred to white 96-well plates for luminescence read (Tecan Infinite 200 Pro). Data are presented as the mean ± SD from three independent experiments.

##### 2.8. Statistical analysis

Statistical analyses were performed using GraphPad Prism v5.01. Data were collected in triplicate from at least three independent experiments. The results were shown as mean ± standard deviation (SD). The half-maximal inhibitory concentrations (IC_50_) were calculated by fitting experimental values to a sigmoidal bell-shaped equation. Differences between the means of treatments with free DMU-214 and three DMU-214 liposomal formulations were evaluated using the Student *t*-test. The level of statistical significance was taken as *p* < 0.05.

## 3. Results and discussion

### 3.1. DMU-214-loaded liposomes characteristics

Several studies have demonstrated that entrapment of *trans-*stilbene derivatives increases aqueous solubility and further bioavailability (Zu et al., 2018). With the log P of 3.1, RSV is a lipophilic compound, while its derivative DMU-214 characterized by logP of 3.5 – practically insoluble in water – even more. Therefore, DMU-214, as a water-insoluble substance, requires a solubilization strategy to obtain pharmaceutical formulation for a feasible administration route in any potential therapeutic applications. DMU-214-loaded liposomes containing different structural phospholipids (DMPC, DPPC or POPC) were characterized in this work. Three formulations were obtained for each structural phospholipid: blank formulation and embedding DMU-214 at initial drug-to-lipid molar ratios (D/L) 0.1 and 0.3 (see Table 1).

**Table 1.** Composition of studied liposomes: structural phospholipids, samples name, components, initial molar ratio and initial drug-to-lipid (D/L) molar ratios.

Structural phospholipid	Sample name	Components	Initial molar ratio	Initial D/L molar ratio
DMPC 14:0 PC	DMPC blank	POPG/DMPC	2/8	–
	DMU-214/DMPC 0.1	DMU-214/POPG/DMPC	1/2/8	0.1
	DMU-214/DMPC 0.3	DMU-214/POPG/DMPC	3/2/8	0.3
DPPC 16:0 PC	DPPC blank	POPG/DPPC	2/8	–
	DMU-214/DPPC 0.1	DMU-214/POPG/DPPC	1/2/8	0.1
	DMU-214/DPPC 0.3	DMU-214/POPG/DPPC	3/2/8	0.3
POPC 16:0-18:1 PC	POPC blank	POPC/DMPC	2/8	–
	DMU-214/POPC 0.1	DMU-214/POPG/POPC	1/2/8	0.1
	DMU-214/POPC 0.3	DMU-214/POPG/POPC	3/2/8	0.3

**Figure 1.**
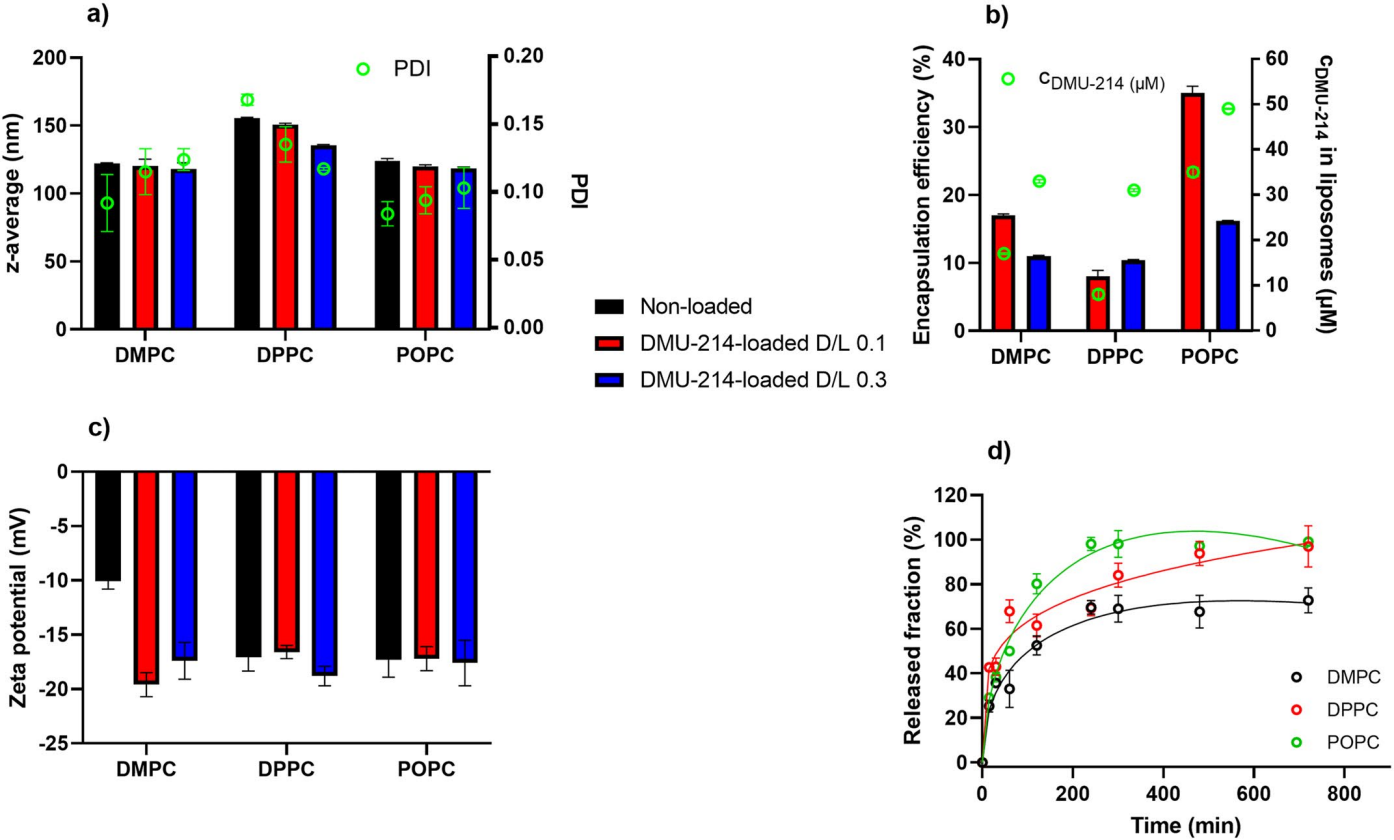
Characteristics of DMU-214 loaded liposomes and non-loaded analog formulations: a) liposome size expressed as z-average (nm) and polydispersity index (PDI), b) DMU-214 encapsulation efficiency (%) and concentration in liposomal formulations (µM), c) zeta potential (mV), d) *in vitro* release profiles of DMU-214-loaded formulations at 0.3 D/L ratio – open circles represent experimentally determined values, lines – correspond to data predicted from Peppas-Sahlin mathematical model. Standard deviations were obtained from measurements (a, b, c) or experiments (d) repeated three times.

Results of DLS measurements are presented in Figure 1a. DMPC- and POPC-based liposomes were smaller than those of corresponding DPPC-based formulations. The z_av_ of DMU-214-loaded liposomes was around 118.0-124.0 nm for DMPC- and POPC-based liposomes and 135.5-155.5 for DPPC liposomes. Incorporation of DMU-214 into liposomes did not affect much the z_av_ of liposomes irrespectively of D/L applied, except DMU-214-loaded at 0.1 and 0.3 D/L ratios. The determined PDI values (0.084-0.168) reflected the homogenous size distribution of particles in the studied samples. PDI increased with increasing DMU-214 loading in DMPC and POPC formulations, but the difference was statistically significant only for DMPC blank and DMU-214/DMPC 0.3 formulations. In the case of DPPC-family carriers, PDI decreased with increasing content of incorporated DMU-214.

The encapsulation efficiency of DMU-214 did not exceed 50% (Figure 1b). Liposomes containing less saturated POPC had greater encapsulation efficiency (35.0% for D/L 0.1 and 16.2% for D/L 0.3) than the more saturated DMPC and DPPC formulations. In the DMU-214/DMPC and DMU-214/POPC liposomes, significantly higher encapsulation efficiency was observed for an initial D/L of 0.1. However, the highest concentration of DMU-214 (c = 49 µM) was determined in DMU-214/POPC 0.3 liposomes, while relatively comparable DMU-214 concentrations of 33, 31 and 35 µM were calculated in

DMU-214/DMPC 0.3, DMU-214/DPPC 0.3 and DMU-214/POPC 0.1, respectively. DMU-214, as a hydrophobic drug, partitions into the lipophilic compartment of the bilayer. Unsaturated phospholipids (such as POPC or POPG) are known to form a less densely packed membrane. Therefore, a liposomal membrane that contains more unsaturated phospholipids would have more intermolecular spaces in which DMU-214 molecules could be incorporated (Kulkarni et al., 1995; Tim Leaver et al., 2018).

The colloidal stability of liposomes in aqueous suspension is governed mainly by their surface properties (Taha et al., 2020). Zeta potential describes the colloidal stability of nanocarriers and plays a vital role in understanding nanocarrier surface interactions affecting cellular uptake, biodistribution, and bioavailability (Kumar & Dixit, 2017). The magnitude of the zeta potential is predictive of the colloidal stability of the solution (Taha et al., 2020). Zeta potentials of DMU-214-loaded liposomes were in the range of −16.6 and −19.6 mV (Figure 1c), corresponding to quite good stability. The negative charge of the liposomal membrane results from the presence of POPG, an anionic phospholipid. Dispersions with greater than −25 mV zeta potential may tend to form agglomerates due to van der Waals, hydrophobic interactions, and hydrogen bonding (Kumar & Dixit, 2017). Noteworthy, agglomeration can be overcome during further development by incorporating phospholipids functionalized with polyethylene glycol chains, creating steric hindrance on the surface of vesicles. The stability of DMU-214-loaded liposomes defined in terms of their ability to maintain the size over 30 days showed that both diameters size and PDI did not differ more than 10% of the initial values presented in Figure 1a.

Three samples of DMU-214-loaded liposomes (DMPC, DPPC, POPC) with an initial D/L ratio of 0.3 were selected for the release study. The cumulative released fraction of DMU-214 was calculated for each selected liposomal formulation (Figure 1d). 50% of DMU-214 was released within 34, 48, and 102 minutes from DPPC, POPC and DMPC formulations. During the experiment, not more than 70% of DMU-214 was released from DMPC-based liposomes, while *ca.* 100% was released from the samples which contained DPPC or POPC as structural phospholipids. After 4 h, DMU-214 release profiles for DMPC and POPC formulations reached the plateau, while for DPPC liposomes, DMU-214 was continuously released over 12 h.

Subsequently, the experimental data were fitted to the Higuchi, Korsmeyer-Peppas, and Peppas-Sahlin mathematical models using DDSolver software (Zhang et al., 2010). According to the data presented in Table 2, Figure 1d, the experimental data best fit the Peppas-Sahlin model for which the highest r^2^ and the lowest Akaike information criterion (AIC) parameters were obtained compared to other models (Table 2). That briefly means that the release of DMU-214 from the studied liposomal formulations is governed by the contribution of two mechanisms, Fickian diffusion and liposome relaxation (Bruschi, 2015).

**Table 2.** Parameters were obtained by fitting the cumulative release profile of DMU-214 from liposomal formulations to three mathematical models.

Model			
*Parameter*	*Formulation*		
	DMU-214/DMPC 0.3	DMU-214/DPPC 0.3	DMU-214/POPC 0.3
**Higuchi**			
kH	3.477	4.472	4.882
R^2^	0.711	0.616	0.738
AIC	67.7	73.6	73.8
T_50_ (min)	207	125	105
**Korsmeyer-Peppas**			
kKP	14.380	23.013	18.033
n	0.259	0.220	0.278
R^2^	0.945	0.964	0.923
AIC	54.8	54.3	64.8
T_50_ (min)	124	34	39
**Peppas-Sahlin**			
k_1_	7.162	21.626	6.752
k_2_	−0.177	2.7	−0.11
m	0.474	0.179	0.557
R^2^	0.973	0.964	0.988
AIC	50.5	56.3	50.4
T_50_ (min)	102	34	48

$M_{\infty }$ is the amount of drug at the equilibrium state (sometimes very close to the amount of drug contained in the dosage form at the beginning of the release process); $M_{t}$ is the amount of drug released over time ***t***; $k_{H}$,$k_{1,}k_{2}$ – are the release constant of Higuchi, Korsmeyer-Peppas and Peppas-Sahlin, and are constants of incorporation of structural modifications and geometrical characteristics of the system; n,m is the exponent of release related to the drug release mechanism in function of time t; **R** – correlation coefficient; **AIC** - Akaike information criterion; **T_50_** - time at which 50% of a substance is released [Bruschi 2015].

### 3.2. Effect of DMU-214 in liposomal carriers on cells viability

**Figure 2.**
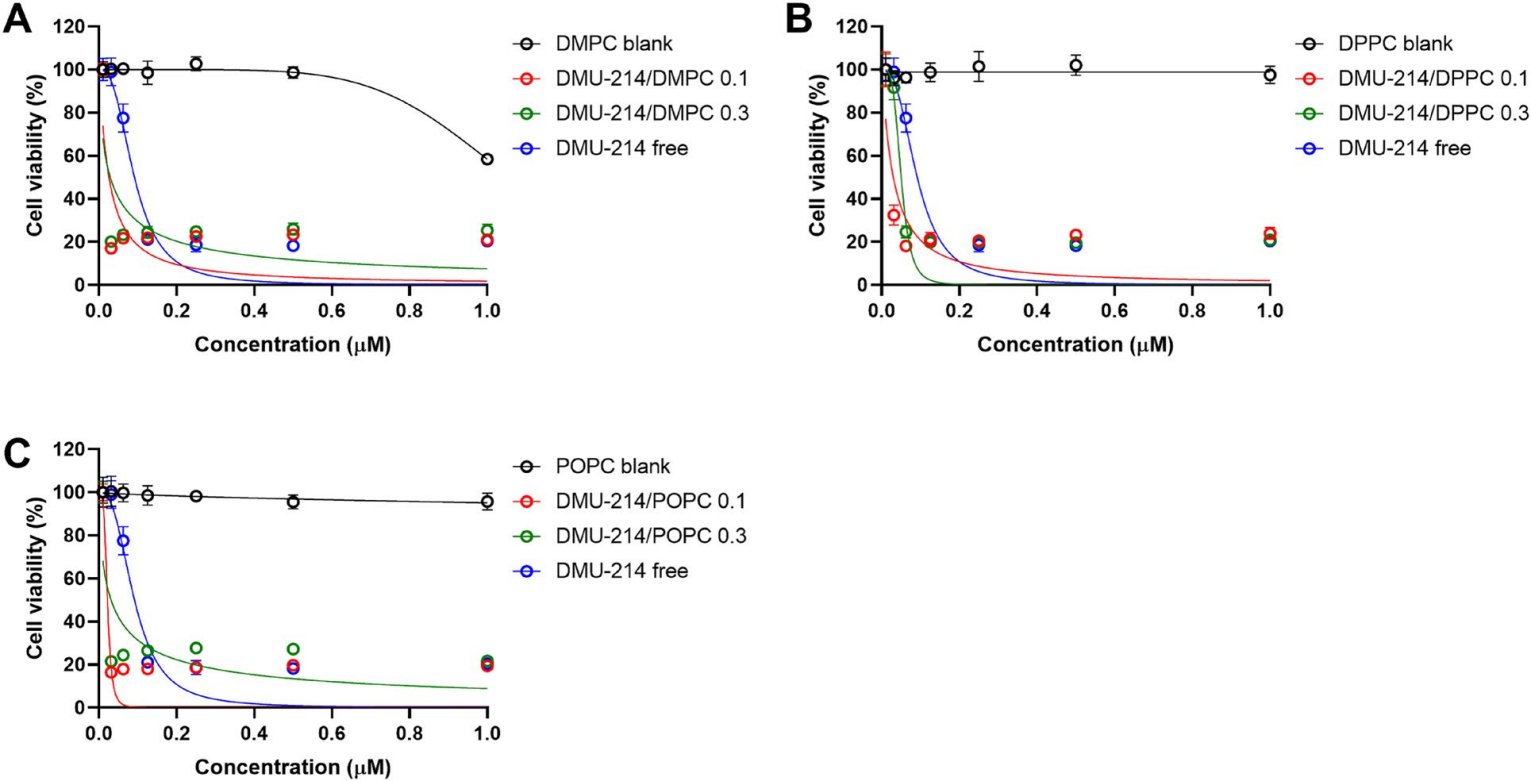
Effect of free DMU-214 and DMU-214 loaded into three liposomal formulations DMPC (A), DPPC (B) and POPC (C) on the SK-OV-3 monolayer cultured cells. DMPC/DPPC/POPC blank: liposomes with DMPC/DPPC/POPC but without DMU-214, diluted by the same dilution factor as the liposomal formulations. The viability was measured using the MTT test after 72 h of drug exposure. IC_50_ values were calculated following a normalized dose-response inhibition curve fitting. Data represent mean values from three independent experiments ± SD.

**Figure 3.**
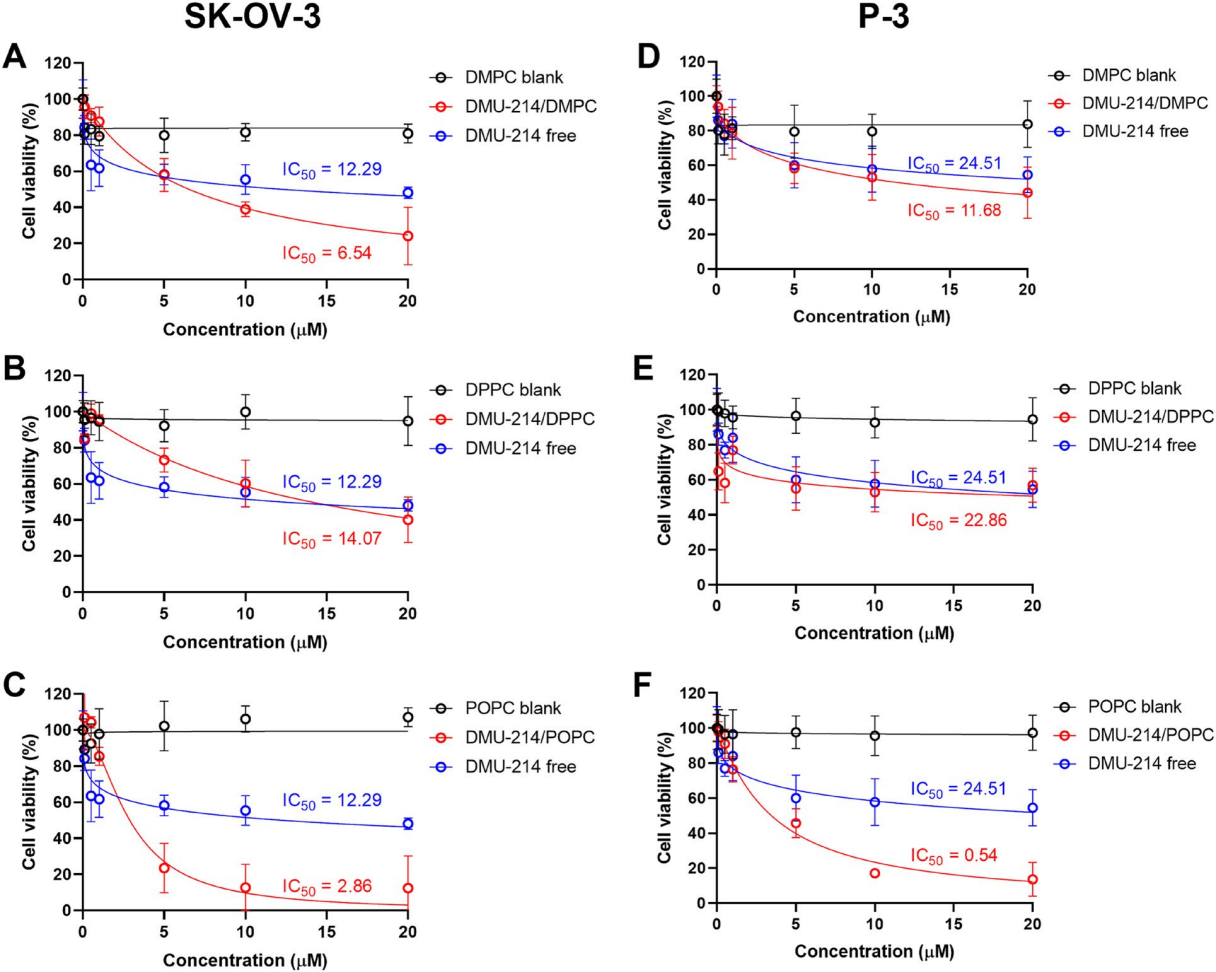
Effect of free DMU-214 and DMU-214 loaded into three liposomal formulations DMPC (A, D), DPPC (B, E), POPC (C, F) on the SK-OV-3 and P-3 patient-derived spheroids. DMPC/DPPC/POPC blank: liposomes with DMPC/DPPC/POPC but without DMU-214, diluted by the same dilution factor as the liposomal formulations. Spheroid viability was measured using CellTiter Glo 3 D after 72 h exposure to the drugs. IC_50_ values were calculated following a normalized dose-response inhibition curve fitting. Data represent mean values from three independent experiments ± SD.

Piotrowska-Kempisty et al. (Piotrowska et al., 2012; 2013) reported a successful preclinical application of 3,4,5,4′-tetramethoxystilebene (DMU-212) in ovarian cancer therapy. Since 3′-hydroxy-3,4,5,4′-tetramethoxystilbene (DMU-214), one of the metabolites of DMU-212 biotransformation, was hardly explored as a drug against ovarian cancer, in this study, we focused on novel liposome-loaded DMU-214 formulations which were evaluated for their biological activity against commercial and patient-derived ovarian cancer cell lines. To investigate the cytotoxic effects of either free DMU-214 or loaded into three liposomal formulations (DMPC, DPPC, POPC) in 2 D cell culture (SK-OV-3) and 3 D tumor spheroids (SK-OV-3 and P-3 patient-derived cell culture), an MTT and CellTiter Glo 3 D cell viability assays were performed after 72 h. DMU-214 was used at concentration ranges of 0-1 µM for monolayer cell culture and 0-20 µM for spheroids culture. The results of cytotoxicity activity of liposome-loaded DMU-214 formulations are listed in Table 3 and presented in Figures 2 and 3.

**Table 3.** The IC_50_ values (µM) for free DMU-214 and its liposomal formulations in 2D SK-OV-3 as well as 3D (spheroids) SK-OV-3 and patient-derived (P-3) cell culture models.

**IC_50_** **Model**	DMU-214 free	DMU-214/DMPC		DMU-214/DPPC		DMU-214/POPC	
		0.1	0.3	0.1	0.3	0.1	0.3
SK-OV-3 2 D	0.092 ±0.004	0.026 ±0.0006	0.029 ±0.0008	0.030 ±0.001	0.050 ±0.002	0.022 ±0.0005	0.031 ±0.0007
*p value*	*Reference value*	*<0.0001* ****	*<0.0001* ****	*<0.0001* ****	*<0.0001* ****	*<0.0001* ****	*<0.0001* ****
SK-OV-3 3 D	12.29 ± 1.1	NA	6.54 ± 0.7	NA	14.07 ± 1.3	NA	2.86 ± 0.3
*p value*	*Reference value*	NA	*0.0007* ***	NA	*0.1323* *ns*	NA	*0.0001* ***
P-3 3 D	24.51 ± 2.6	NA	11.68 ± 1.4	NA	22.86 ± 2.5	NA	0.54 ± 0.04
*p value*	*Reference value*	*NA*	*0.0017* **	*NA*	*0.4725* *ns*	*NA*	*<0.0001* ****

*The IC_50_ values were calculated by fitting experimental values to a sigmoidal bell-shaped equation. Each drug concentration was made in triplicate in each experiment, and the final obtained IC_50_ represents the mean and standard deviation of three separate experiments. Each DMU-214 liposomal formulation was compared to free DMU-214 by Student t-test. NA – not applicable; ns – no significance;* ^**^, ^***^, ^****^ *- statistical significance*.

#### 3.2.1. 2D cell culture

The obtained results showed that all three liposomal formulations of DMU-214 at two drug-to-lipid molar ratios, 0.1 and 0.3, are more potent (IC_50_ values in the range 22-50 nM) than the non-loaded DMU-214 (DMU-214 free) in the investigated SK-OV-3 ovarian cancer cell line (IC_50_ = 92 nM), (Table 3). Our results indicated that the liposome-encapsulated DMU-214 was 2-4 times more effective than free DMU-214. In addition, we found statistically significant differences in IC_50_ values for our 2 D model of SK-OV-3 cells treated with all three liposomal formulations of DMU-214 compared to free DMU-214 formulation (*p* < 0.0001 for all formulations tested).

It is worthy to notice that the liposomal delivery system possessed highly favorable performance since it did not cause any toxicity by itself but was very effective in the delivery of DMU-214 for the treatment of ovarian cancer. The one exception was the DMPC blank, which has been shown to exert cytotoxicity in the SK-OV-3 cell line (Figure 2). However, only the highest concentrations of DMPC have decreased cell viability. Concomitantly, DMU-214/POPC formulation disclosed the highest therapeutic potency in SK-OV-3 cells, making this liposomal formulation of DMU-214 therapeutically promising in fighting malignancy. Several studies showed that cancer stem-like cells (CSCs) are present in the SK-OV-3 cell line (Ma et al., 2010). Like with other cancers, the presence of a subpopulation of cancer stem-like cells has been associated in ovarian cancer with chemoresistance and tumor relapse (Sánchez-García et al., 2007). CSCs were also found to be resistant to anticancer drugs and irradiation. Thus, CSCs might be a crucial target for DMU-214 therapy. In this study, we employed the SK-OV-3 cell line because it has been widely used for ovarian cancer studies and forms non-adherent spheres with remarkable stem cell properties. Likewise, the patient-derived spheroids retain the biological characteristics of clinical cancers and are instrumental in understanding the chemoresistance of ovarian cancer cells. However, our obtained P-3 patient-derived cell line originally derived as a spheroid culture could not adapt to the adherent culture in a swift. Therefore, we tested the biological activity of the liposomal DMU-214 formulations only in SK-OV-3 monolayer culture.

#### 3.2.2. 3D cell culture

**Figure 4.**
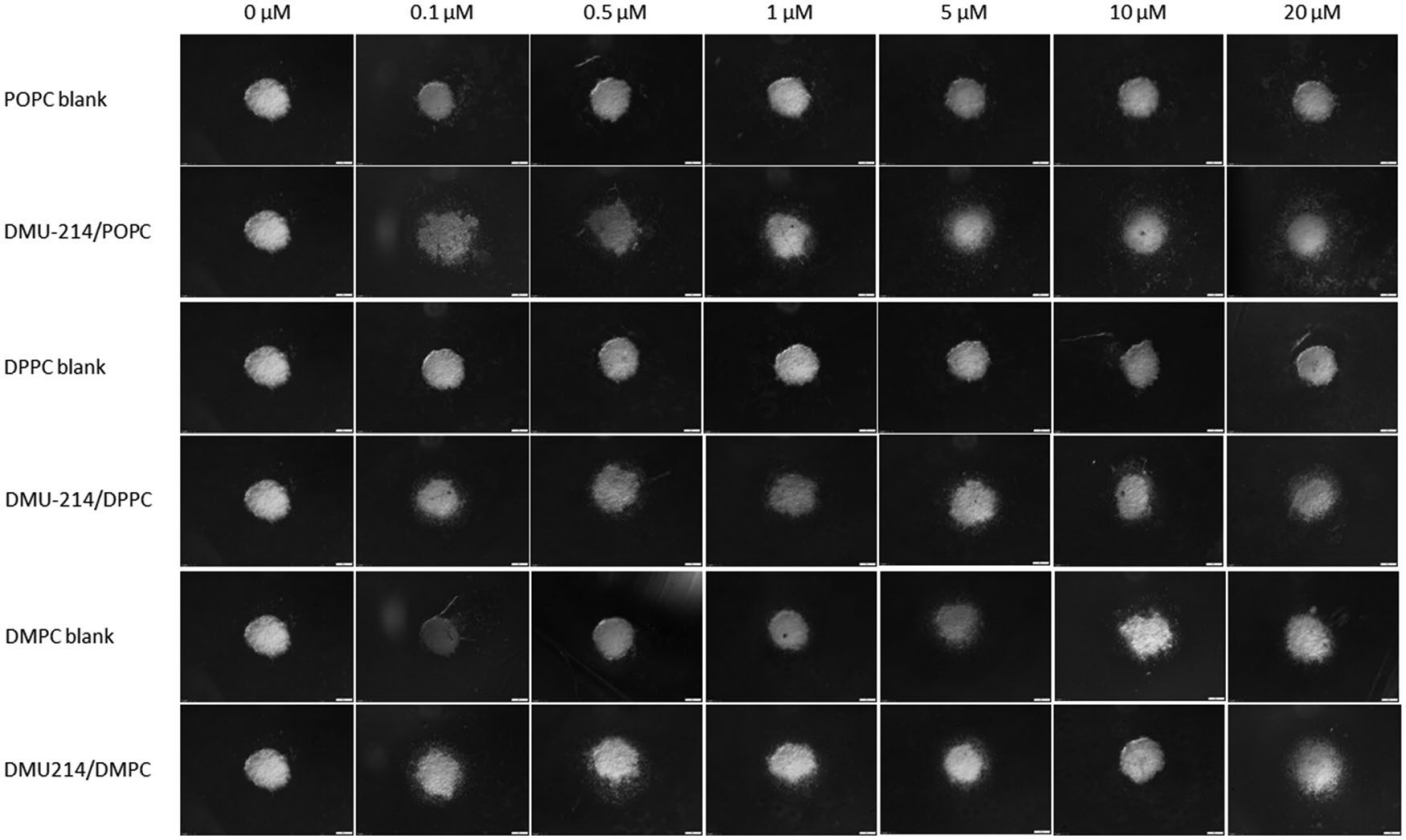
Spheroids growth and morphology upon treatment with DMU-214 liposomal formulations (DMPC/DPPC/POPC). Spheroids were pre-formed for 7 days with 5 000 SK-OV-3 cells and treated with the indicated concentrations of DMU-214 formulations. The images were acquired 72 hours after administration of the formulation with the Olympus ix73 inverted microscope.

Having established the optimal conditions for spheroid assembly, we examined the effect of liposomal formulations of DMU-214 on preformed SK-OV-3 and P-3 patient-derived spheroids. The use of 3 D cell cultures, such as spheroids, would allow greater predictability of efficacy and toxicity in humans before drugs move into clinical trials (Yamada & Cukierman, 2007). Based on the results presented in Table 3, DMU-214-loaded liposomes with the highest concentration of DMU-214, which corresponds to DMU-214-loaded at 0.3 D/L ratios for individual phospholipids (DMPC, DPPC or POPC), were chosen for further investigation in 3 D cell culture models. As shown in Table 3 and Figure 3, all DMU-214 formulations exhibited cytotoxic activities in both spheroid models. The determined IC_50_ values for DMU-214-loaded liposomes were between 2.86–14.07 μM and 0.54–22.86 μM in the SK-OV-3 and P-3 patient-derived spheroids respectively. In addition, IC_50_ values of liposomal formulations of DMU-214 excluding DMU-214/DPPC had statistically significant differences from those of free DMU-214. Considering the therapeutic potency of examined DMU-214 liposomal formulations in fighting ovarian cancer cells in 3 D models, it should be noted that IC_50_ values of two liposomal formulations of DMU-214, DMPC- and POPC-based, exceeded the therapeutic potency of free DMU-214. Since DMU-214 liposomal formulations exert more potent cytotoxic activity in both examined spheroid models than the free DMU-214 (SK-OV-3 IC_50_ = 12.29 μM; P-3 IC_50_ = 24.51 μM), they might be suggested as potential chemotherapeutics in ovarian cancer. In this series of phospholipid formulations, the only exception was a DMU-214/DPPC whose cytotoxicity in SK-OV-3 spheroids investigated was slightly lower than that of free DMU-214. Moreover, most DMU-214 formulations showed quite higher activity against spheroids from commercially available ovarian cancer cells SK-OV-3 compared to P-3 patient-derived spheroids. Figure 4 shows morphological changes of

SK-OV-3 spheroids after all liposomal formulations treatment. A significant decrease in the thickness of the proliferative zones was observed. Furthermore, detaching cells from the

SK-OV-3 spheroids were also noted.

It can be noted that type of phosphatidylcholine used in liposomes affected the cytotoxicity of DMU-214 both in 2 D and 3 D cellular models (Table 3). Formulations containing phosphatidylcholine characterized by lower transition temperature (POPC, T_m_= −2 °C) and thus forming more flexible vesicles usually exerted a higher cytotoxic effect than those with DMPC (T_m_= 24 °C) or DPPC (T_m_= 41 °C). Importantly, DMU-214/POPC formulation was highly active against P-3 patient-derived spheroids, reaching the IC_50_ of 0.54 ± 0.04 µM. The higher cytotoxicity of fluidic liposomes, resulting from higher cellular uptake, was demonstrated by Chen et al. (Chen et al., 2016). The phospholipid composition of the liposomal membrane was proved to influence the rate of liposome uptake and internalization by cells (Dini et al., 1998; Chen et al., 2016). It was suggested that it was easier for a fluidic liposomal membrane to fuze with the membrane of cells (Chen et al., 2016).

The great advantage of a patient-derived 3 D model, compared to other 3 D systems, is that it more accurately reflects *in vivo* biology. One of the important features of patient-derived organoids is that they are genetically and phenotypically more similar to a native tumor, forming intratumoral heterogeneity and drug resistance (Nanki et al., 2020).

Recently, it was revealed that the type of model used was found to play an important role in the sensitivity of cell lines to tested drugs, with 3 D spheroid models being less sensitive than their 2 D monolayer counterparts (Dunn et al., 2022). Of note, in monolayer culture, SK-OV-3 cells have displayed a marked difference in the sensitivity to free DMU-214 and its liposomal formulations since the IC_50_ values were in the nanomolar range of 22-50 nM. Cell viability in the 3 D model was substantially higher than in the 2 D model, which may be related to the drug formulations being less able to penetrate these spheroids due to their density. The large size of the spheroids likely resulted in a hypoxic, nutrient-deprived environment toward the center, and, therefore, the central cells are unlikely to be alive. Increases in the size of ovarian cancer cell line spheroids have been demonstrated to reduce their sensitivity to cytotoxic agents (Gunay et al., 2020), likely to the formation of necrotic and quiescent regions, resulting from the increased hypoxic environment and reduced drug penetration compared to 2 D (Hirschhaeuser et al., 2010). On the other hand, observed chemoresistance could be attributed to a slower proliferation rate within spheroids, environmental changes in 3 D spheroids, and possible change in cellular phenotype of ovarian cancer cells in three-dimensional culture compared to 2 D (Correia & Bissell, 2012). Despite the above considerations, using 3 D aggregate models (spheroids, organoids, patient-derived ones) over 2 D monolayer systems is important in investigating the preclinical efficacy of liposomal formulations of DMU-214.

## 4. Conclusions

In the present study, DMU-214-loaded liposomes were developed for the first time. Building on 3 D *in vitro* model, we used the patient-derived tumor model of ovarian cancer to assess drug responses. Such a preclinical model more accurately reflects *in vivo* biology and can predict clinical outcomes. Noteworthy, the choice of phosphatidylcholine used in the preparation of liposomal formulations affected DMU-214 encapsulation efficacy and release rate and DMU-214 cytotoxicity in ovarian cancer models. The highest antiproliferative activity was observed for formulation composed of phosphatidylcholine (POPC) with the lowest transition temperature, forming more flexible vesicles. We showed that the liposomal DMU-214 improved cytotoxic activity against ovarian cancer cell line SK-OV-3 and patient-derived ovarian cancer spheroids, decreasing the IC_50_ value compared to free DMU-214. Overall, the results showed that studied DMU-214-loaded liposomal formulations (especially DMU-214/POPC) potentially represent a promising and more effective delivery strategy for DMU-214, warranting further investigation for the treatment of ovarian cancer.

## Author contributions

Andrzej Nowicki: Investigation, Data curation, Formal analysis, Writing – original draft; Dariusz Wawrzyniak: Investigation, Methodology, Formal analysis, Writing – original draft; Mikolaj Czajkowski: Investigation; Małgorzata Józkowiak: Investigation; Michał Pawlak: collecting cancer tissue samples; Marcin Wierzchowski: synthesis of DMU-214; Katarzyna Rolle: Formal analysis; Paulina Skupin-Mrugalska: Investigation, Data curation, Methodology, Writing – original draft; Hanna Piotrowska-Kempisty: Data curation, Formal analysis, Investigation, Methodology, Writing - review & editing.
